# Supplementary figures and images for: Multiple myeloma: Combination therapy of BET proteolysis targeting chimeric molecule with CDK9 inhibitor
Source: PLoS One. 2020 Jun 19;15(6):e0232068. doi: 10.1371/journal.pone.0232068 (PMC7304913; doi:10.1371/journal.pone.0232068)

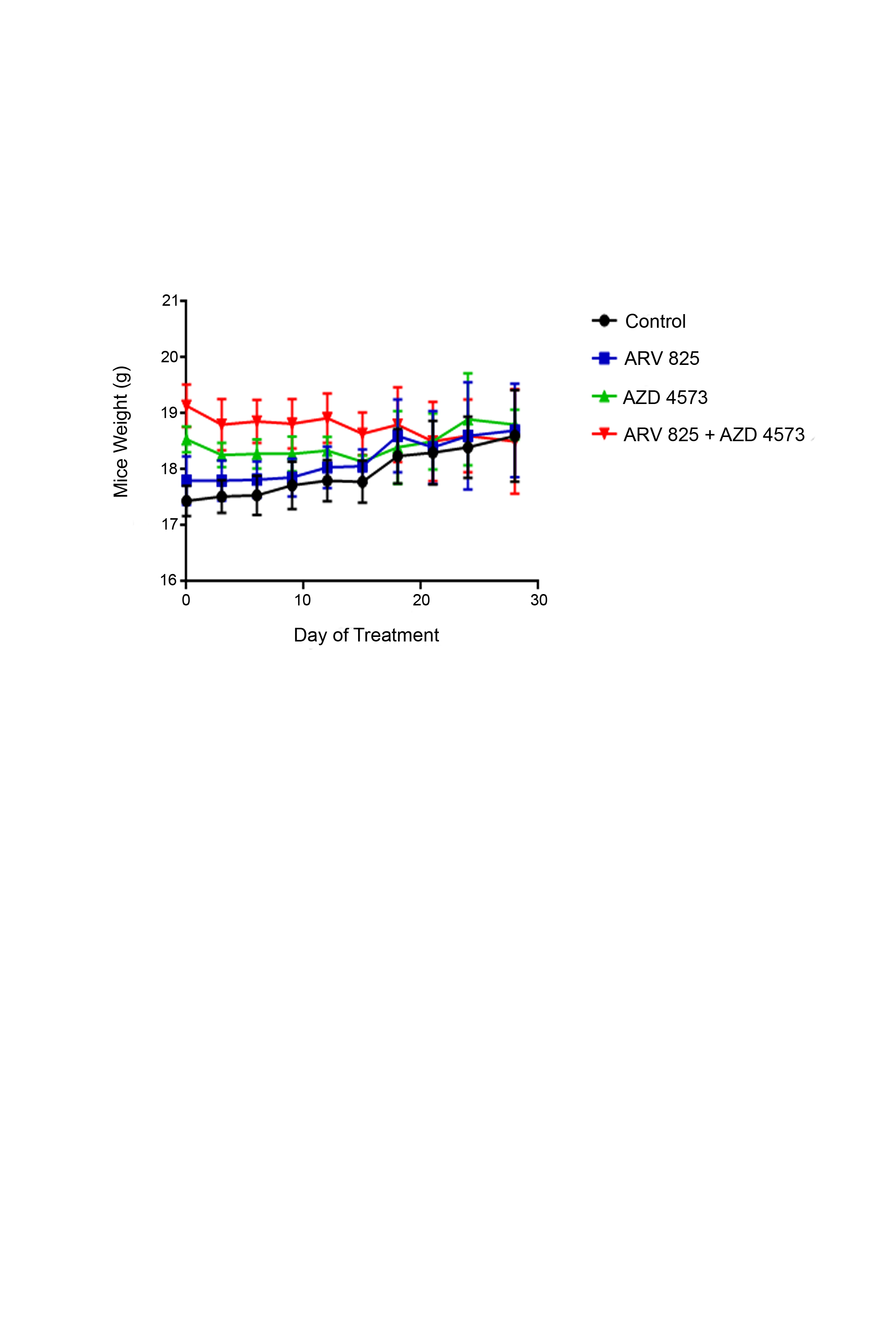

Supplement: S1 Fig — Comparison of weight of mice after treatment with ARV 825 (5 mg/kg IP daily for 28 days), AZD 4573 (10 mg/kg, IP, twice a day with 2 h interval for two consecutive days/week for 4 weeks), combination of both drugs or diluent control. Mean ± SD of 5 mice in each group. (TIF) [file pone.0232068.s001.tif]

KMS11

Figure 4A

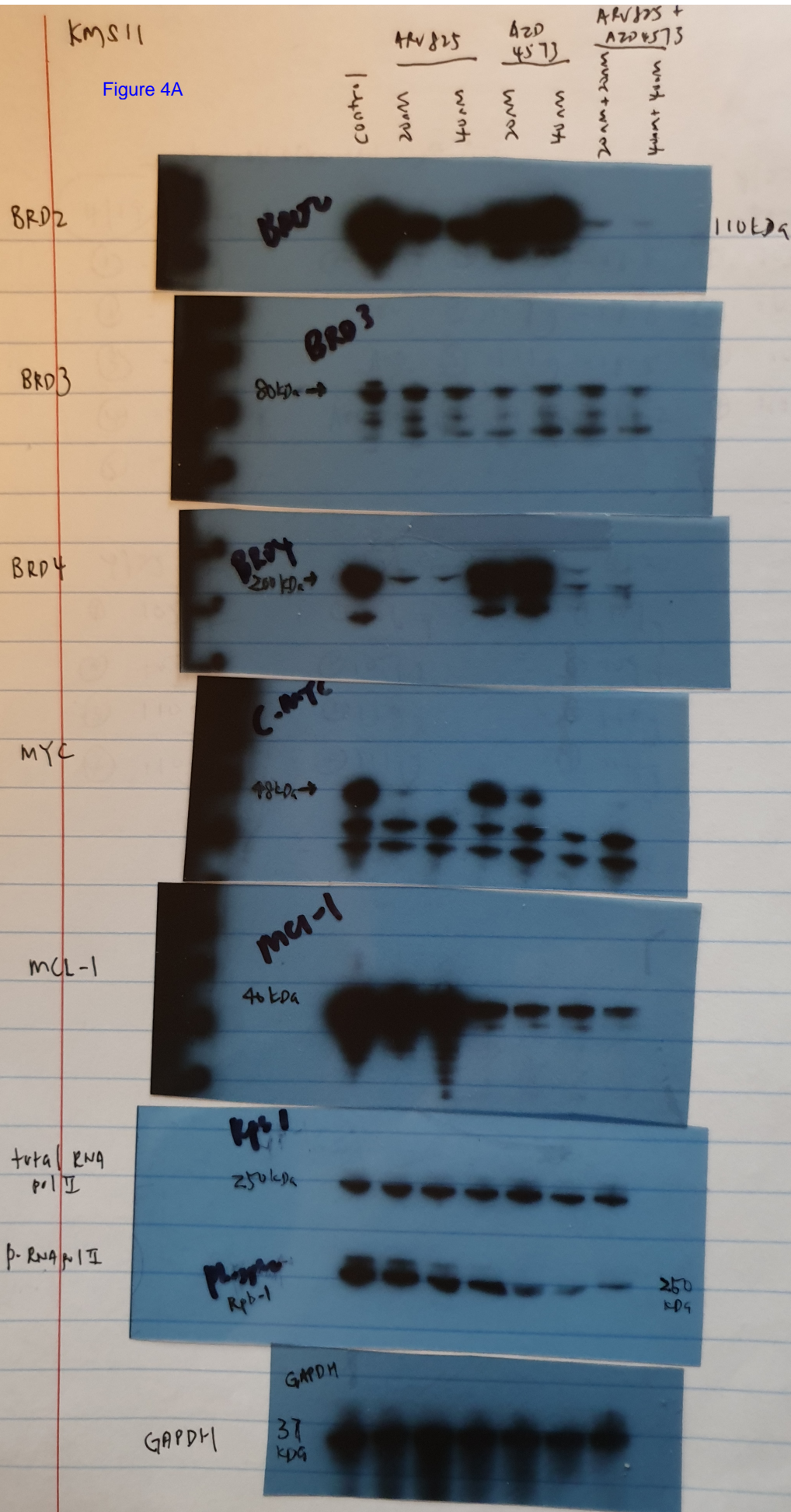

8226

Figure 4B

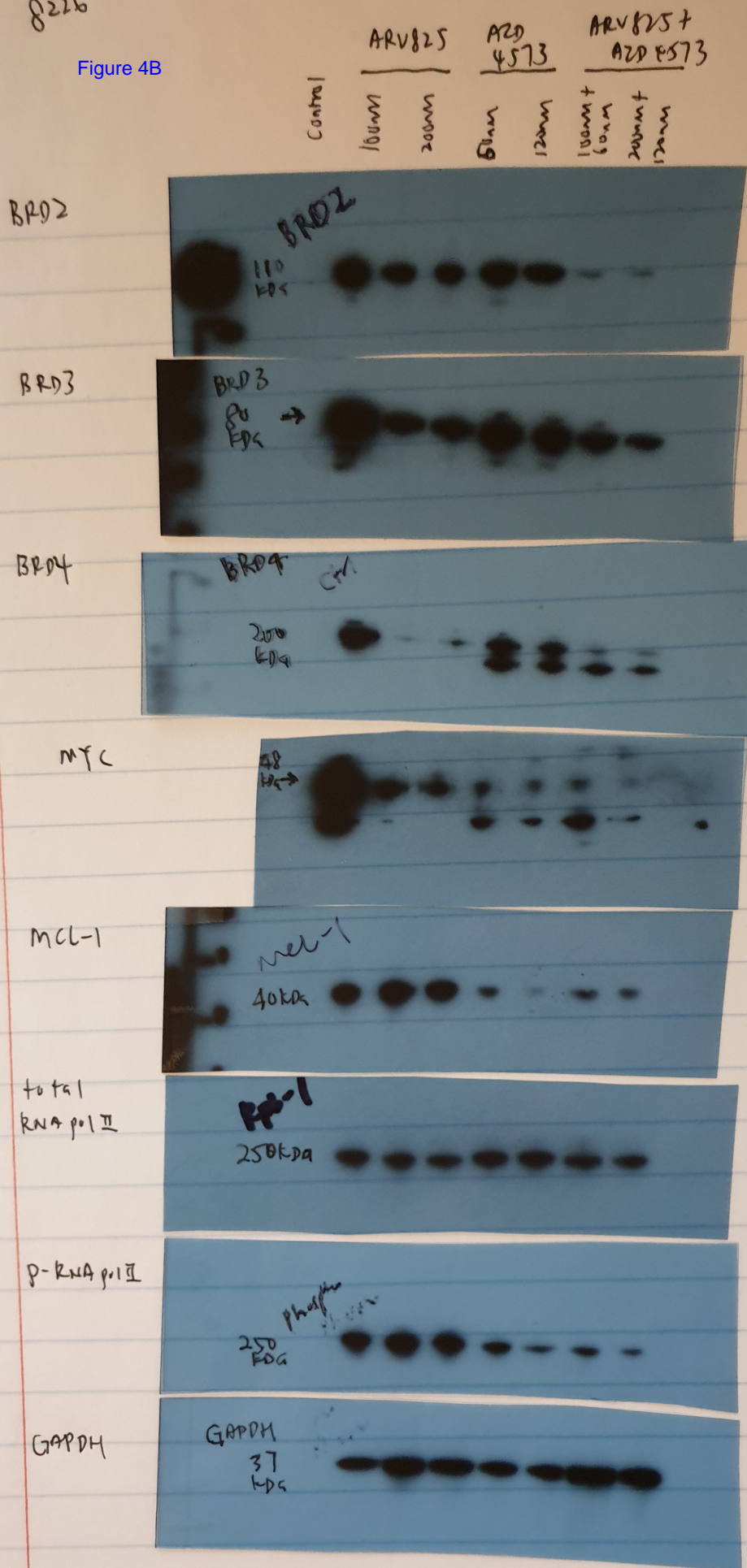

Supplement: S1 Raw images — (PDF) [file pone.0232068.s002.pdf]
