# Supplementary material for: Multiple myeloma: Combination therapy of BET proteolysis targeting chimeric molecule with CDK9 inhibitor
Source: PLoS One. 2020 Jun 19;15(6):e0232068. doi: 10.1371/journal.pone.0232068 (PMC7304913; doi:10.1371/journal.pone.0232068)
Supplement: S1 Table — (DOCX) [file pone.0232068.s003.docx]

**S1 Table. List of antibodies**

| **List of antibodies** | |
| --- | --- |
| **Antibodies** | **Manufacturer** |
| BRD 2 | Cell Signaling Technology, 5848 |
| BRD 3 | Proteintech, 11859-1-AP |
| BRD 4 | Cell Signaling Technology, 13440 |
| CDK 9 | Cell Signaling Technology, 2316T |
| MCL 1 | Cell Signaling Technology, 5453 |
| Phosphor- Rpb1 CTD (ser2) | Cell Signaling Technology, 13499 |
| Rpb1 CTD | Cell Signaling Technology, 2629 |
|  | |
| **List of inhibitors** | |
| **Inhibitors** | **Manufacturer** |
| AZD 4573 | MedChemExpress, Catalog no. HY-112088 |
| Bortezomib | Selleckchem Catalog no. S1013 |
| Melphalan | Selleckchem Catalog no. S8266 |
