# Supplementary material for: Multiple myeloma: Combination therapy of BET proteolysis targeting chimeric molecule with CDK9 inhibitor
Source: PLoS One. 2020 Jun 19;15(6):e0232068. doi: 10.1371/journal.pone.0232068 (PMC7304913; doi:10.1371/journal.pone.0232068)
Supplement: S2 Table — (DOCX) [file pone.0232068.s004.docx]

**S2 Table. IC50s of AZD 4573 against MM cells, 72 h**

| **Cell lines** | **AZD 4573 IC50s ± SD (nM), 72 h** |
| --- | --- |
| MM1S (14:16) | 8 ± 0.6 |
| KMS11 (4:14) | 8 ± 0.1 |
| MM1S res (lenalidomide resistant) | 8 ± 0.4 |
| MM1R (steroid resistant) | 8 ± 3.0 |
| KMS11 res (lenalidomide resistant) | 9 ± 1.1 |
| KMS18 | 12 ± 1.1 |
| KMS28 (4:14) | 17 ± 1.4 |
| KMS12BM | 21 ± 2.1 |
| H929 | 20 ± 0.3 |
| 8226 LR5 (Melphalan resistant) | 22 ± 3.1 |
| 8226 (14:16) | 23 ± 3.2 |
| 8226 P100V (bortezomib resistant) | 70 ± 4.4 |
|  | |
| **Cell lines** | **MC180295 IC50s ± SD (nM), 72 h** |
| H929 | 260 ± 20 |
| KMS11 | 280 ± 23 |
| KMS18 | 330 ± 24 |
| KMS11 res (lenalidomide resistant) | 330 ± 40 |
| MM1S | 340 ± 42 |
| MM1S res (lenalidomide resistant) | 340 ± 42 |
| MM1R (steroid resistant) | 350 ± 31 |
| 8226 | 460 ± 43 |
| 8226 LR5 (Melphalan resistant) | 520 ± 22 |
| KMS28 | 510 ± 14 |
| KMS12BM | 700 ± 9 |
| 8226 P100V (bortezomib resistant) | >1000 |
