# Supplementary material for: Multiple myeloma: Combination therapy of BET proteolysis targeting chimeric molecule with CDK9 inhibitor
Source: PLoS One. 2020 Jun 19;15(6):e0232068. doi: 10.1371/journal.pone.0232068 (PMC7304913; doi:10.1371/journal.pone.0232068)
Supplement: S3 Table — (DOCX) [file pone.0232068.s005.docx]

**S3 Table. IC50s of ARV 825 against MM cells, 72 h**

| **Cell lines** | **ARV 825 IC50 ± SD (nM), 72 h** |
| --- | --- |
| KMS11 | 9 ± 1.9 |
| MM1R (Steroid resistant) | 10 ± 1.8 |
| KMS12BM | 11 ± 1.3 |
| MM1S | 11 ± 1.8 |
| H929 | 16 ± 1.6 |
| KMS18 | 17 ± 1.1 |
| 8226 LR 5 (Melphalan resistant) | 20 ± 1.9 |
| KMS11 res (Lenalidomide resistant) | 70 ± 1.4 |
| U266 | 71 ± 1.8 |
| 8226 | 84 ± 1.4 |
| KMS28BM | 137 ± 1.1 |
| 8226 P100V (Bortezomib resistant) | 500 ± 0.6 |
| MM1S res (Lenalidomide resistant) | >500 |
