# Supplementary material for: Multiple myeloma: Combination therapy of BET proteolysis targeting chimeric molecule with CDK9 inhibitor
Source: PLoS One. 2020 Jun 19;15(6):e0232068. doi: 10.1371/journal.pone.0232068 (PMC7304913; doi:10.1371/journal.pone.0232068)
Supplement: S4 Table — Combination index of AZD 4573 synergistic with ARV 825 (CI < 1, CI = 1 and CI > 1 represent synergism, additive and antagonism, respectively). (DOCX) [file pone.0232068.s006.docx]

**S4 Table. Combination Index.** Combination index of AZD 4573 synergistic with ARV 825 (CI < 1, CI = 1 and CI > 1 represent synergism, additive and antagonism, respectively)

| **KMS11 (cell line)** | | | |
| --- | --- | --- | --- |
|  | **ARV 825** | | |
|  | **2.5 nM** | **5 nM** | **10 nM** |
| **AZD 4573** |  |  |  |
| **2.5 nM** | 0.75 | 0.71 | 0.82 |
| **5 nM** | 0.83 | 0.85 | 1.1 |
|  | | | |
| **8226 (cell line)** | | | |
|  | **ARV 825** | | |
|  | **40 nM** | **80 nM** | **160 nM** |
| **AZD 4573** |  |  |  |
| **12.5 nM** | 0.38 | 0.36 | 0.40 |
| **25 nM** | 0.63 | 0.66 | 0.71 |
|  | | | |
| **KMS28 (cell line)** | | | |
|  | **ARV 825** | | |
|  | **50 nM** | **100 nM** | **200 nM** |
| **AZD 4573** |  |  |  |
| **7.5 nM** | 0.61 | 0.65 | 0.62 |
| **15 nM** | 0.64 | 0.66 | 0.64 |
