# Supplementary material for: Multiple myeloma: Combination therapy of BET proteolysis targeting chimeric molecule with CDK9 inhibitor
Source: PLoS One. 2020 Jun 19;15(6):e0232068. doi: 10.1371/journal.pone.0232068 (PMC7304913; doi:10.1371/journal.pone.0232068)
Supplement: Attachment — Submitted filename: Response to Reviewers.docx [file pone.0232068.s007.docx]

**Rebuttal letter**

1. When submitting your revision, we need you to address these additional requirements.

Please ensure that your manuscript meets PLOS ONE's style requirements, including those for file naming. The PLOS ONE style templates can be found at

https://journals.plos.org/plosone/s/file?id=wjVg/PLOSOne_formatting_sample_main_body.pdf and

<https://journals.plos.org/plosone/s/file?id=ba62/PLOSOne_formatting_sample_title_authors_affiliations.pdf>

Author’s reply:

Thank you for the comment. The revised manuscript is corrected to meet the PLOS ONE’s style requirements.

2. At this time, we request that you please report additional details in your Methods section regarding animal care, as per our editorial guidelines:

(1) Please state the source of the mice used in the study

Author’s reply:

The source of the mice is now included in revised manuscript in Methods section. Line 161-162

(2) Please provide details of animal welfare (e.g., shelter, food, water, environmental enrichment)

Author’s reply:

It is included in revised manuscript in Methods section. Line 161-164

(3) Please describe any steps taken to minimize animal suffering and distress, such as by administering anaesthesia

Author’s reply:

As suggested, it has been included in revised manuscript in Methods section. Line 174-176

(4) Please describe the post-operative care received by the animals, including the frequency of monitoring and the criteria used to assess animal health and well-being.

Author’s reply:

The animal health and well-being were monitored daily by trained staffs at comparative medicine according to the approved IACUC protocol by Cedars Sinai Medical Center.

3. Please provide additional information about each of the cell lines used in this work, including any quality control testing procedures (authentication, characterisation, and mycoplasma testing). For more information, please see <http://journals.plos.org/plosone/s/submission-guidelines#loc-cell-lines>

Author’s reply:

As recommended, the additional information about cell lines used is included in the revised manuscript in Materials and Methods section. Line 89-92. STR analysis was done on all cell lines.

In addition, please provide the source, culture conditions and quality control testing procedures of the HEK 293 FT cells used in your study.

Author’s reply:

The information about 293FT cells is now included in revised manuscript in Materials and Methods section. Line 92-94.

4. In the Methods section, please provide the product number and any lot numbers of the ARV-825 and AZD 4573 purchased from chemical companies for your study.

Author’s reply:

As suggested, the catalog number for both ARV 825 and AZD 4573 are included in revised manuscript in Methods section. Line 130 and 132.

5. To comply with PLOS ONE submission guidelines, in your Methods section, please provide additional information regarding your statistical analyses, including the software used for the analysis. For more information on PLOS ONE's expectations for statistical reporting, please see <https://journals.plos.org/plosone/s/submission-guidelines.#loc-statistical-reporting>.

Author’s reply:

The information regarding statistical analysis is included in revised manuscript in Methods section. Line 180-182.

Reviewer’s comment:

1. Is the manuscript technically sound, and do the data support the conclusions?

The manuscript must describe a technically sound piece of scientific research with data that supports the conclusions. Experiments must have been conducted rigorously, with appropriate controls, replication, and sample sizes. The conclusions must be drawn appropriately based on the data presented.

Reviewer #1: Yes

Author’s reply: Thank you for the comment

2. Has the statistical analysis been performed appropriately and rigorously?

Reviewer #1: Yes

 Author’s reply: Thank you for the comment

3. Have the authors made all data underlying the findings in their manuscript fully available?

The [PLOS Data policy](http://www.plosone.org/static/policies.action#sharing) requires authors to make all data underlying the findings described in their manuscript fully available without restriction, with rare exception (please refer to the Data Availability Statement in the manuscript PDF file). The data should be provided as part of the manuscript or its supporting information, or deposited to a public repository. For example, in addition to summary statistics, the data points behind means, medians and variance measures should be available. If there are restrictions on publicly sharing data—e.g. participant privacy or use of data from a third party—those must be specified.

Reviewer #1: Yes

Author’s reply: Thank you for the comment

4. Is the manuscript presented in an intelligible fashion and written in standard English?

PLOS ONE does not copyedit accepted manuscripts, so the language in submitted articles must be clear, correct, and unambiguous. Any typographical or grammatical errors should be corrected at revision, so please note any specific errors here.

Reviewer #1: Yes

Author’s reply: Thank you for the comment

5. Review Comments to the Author

Please use the space provided to explain your answers to the questions above. You may also include additional comments for the author, including concerns about dual publication, research ethics, or publication ethics. (Please upload your review as an attachment if it exceeds 20,000 characters)

Reviewer #1: Multiple Myeloma: Combination therapy of BET Proteolysis Targeting Chimeric Molecule with CDK9 Inhibitor.
The author reported for the first time that the combination of a BET PROTAC(ARV 825) plus CDK9 inhibitor(AZD 4573) is effective for the treatment of multiple myeloma. It is worthy of publication in PLOS ONE after major revision of the following points.
1. Figure 1 to 6 are not clear enough;
2. Western Blot Analysis needs to be redone.

Author’s reply: Thank you for the comment. The quality of figures has been improved as suggested in revised manuscript.
